# Redox Imbalance and Antioxidant Defenses Dysfunction: Key Contributors to Early Aging in Childhood Cancer Survivors

**DOI:** 10.3390/antiox13111397

**Published:** 2024-11-15

**Authors:** Vanessa Cossu, Nadia Bertola, Chiara Fresia, Federica Sabatini, Silvia Ravera

**Affiliations:** 1 Department of Experimental Medicine, University of Genoa, Via De Toni 14, 16132 Genova, Italy; vanessa.cossu@edu.unige.it; 2 IRCCS Ospedale Policlinico San Martino, Largo Rosanna Benzi, 10, 16132 Genova, Italy; nadia.bertola@hsanmartino.it; 3 UOSD Laboratorio di Terapie Cellulari, IRCCS Istituto Giannina Gaslini, Via Gerolamo Gaslini 5, 16148 Genova, Italy; chiarafresia@gaslini.org (C.F.); federicasabatini@gaslini.org (F.S.)

**Keywords:** aging, antioxidant defenses, childhood cancer survivors, Nrf2, oxidative phosphorylation, oxidative stress, ROS

## Abstract

Survival rates for childhood cancer survivors (CCS) have improved, although they display a risk for early frailty due to the long-term effects of chemo/radiotherapy, including early aging. This study investigates antioxidant defenses and oxidative damage in mononuclear cells (MNCs) from CCS, comparing them with those from age-matched and elderly healthy individuals. Results show impaired antioxidant responses and increased oxidative stress in CCS MNCs, which exhibited uncoupled oxidative phosphorylation, leading to higher production of reactive oxygen species, similar to metabolic issues seen in elderly individuals. Key antioxidant enzymes, namely glucose-6-phosphate dehydrogenase, hexose-6-phosphate dehydrogenase, glutathione reductase, glutathione peroxidase, catalase, and superoxide dismutase, showed reduced activity, likely due to lower expression of nuclear factor erythroid 2–related factor 2 (Nrf2). This imbalance caused significant damage to lipids, proteins, and DNA, potentially contributing to cellular dysfunction and a higher risk of cancer recurrence. These oxidative and metabolic dysfunctions persist over time, regardless of cancer type or treatment. However, treatment with N-acetylcysteine improved Nrf2 expression, boosted antioxidant defenses, reduced oxidative damage, and restored oxidative phosphorylation efficiency, suggesting that targeting the redox imbalance could enhance long-term CCS health.

## 1. Introduction

The increasing survival rates among childhood cancer patients, primarily due to advances in oncological treatments, have led to a growing population of long-term survivors [1,2]. However, this success presents new challenges, as childhood cancer survivors (CCS) are at heightened risk for a spectrum of late effects [3,4,5], including early aging and associated morbidities [6,7,8]. Specifically, young adult CCS exhibit several symptoms associated with aging-related frailty [8,9], such as cardiovascular disease [10,11], diabetes [12], decreased muscle strength [13], reduced physical endurance [14], and cognitive dysfunctions [15,16]. This decline affects not only their physical health but also their overall quality of life (QoL) [17,18].

Several studies suggest that these late effects are not merely a consequence of cancer treatment but also result from a complex interplay of biological processes, including “inflammaging”, a chronic, low-grade inflammation observed as part of the aging process [19,20]. In a recent paper published by our group, we observed that frailty and early aging associated with chemo/radiotherapy may be linked to mitochondrial dysfunction and the consequent increase in oxidative stress [21]. Our study found that CCS aged 1 to 40 years exhibit impaired oxidative phosphorylation (OxPhos), increased reactive oxygen species (ROS) production, and a metabolic shift from aerobic to anaerobic energy production, similar to that observed in healthy individuals over 60 years of age, confirming the accelerated aging process in CCS [21].

In healthy individuals, increased oxidative stress is typically countered by a robust antioxidant defense system that prevents the accumulation of oxidative damage [22,23]. However, these defenses weaken with age [24], as suggested by the oxidative damage theory of aging [25]. Cells utilize various antioxidant systems to mitigate oxidative stress and protect cellular structures [26,27]. Among these, the glutathione pathway is one of the most critical for neutralizing ROS and preserving cellular integrity [28]. Central to this pathway is glutathione (GSH), a tripeptide that acts as a major intracellular antioxidant by directly scavenging free radicals and serving as a substrate for various antioxidant enzymes [29]. The enzyme glutathione reductase (GR) regenerates GSH from its oxidized form, glutathione disulfide (GSSG), maintaining the high GSH/GSSG ratio necessary for effective cellular defense [30]. Additionally, glutathione peroxidase (GPx) uses GSH to reduce hydrogen peroxide and lipid peroxides to water and corresponding alcohols, further detoxifying harmful ROS [31].

The efficiency of the glutathione pathway is also influenced by the availability of NADPH [32], produced via the pentose phosphate pathway, which is essential for the reduction of GSSG to GSH by GR [32]. Glucose-6-phosphate dehydrogenase (G6PD) and hexose-6-phosphate dehydrogenase (H6PD) play a pivotal role in this process by catalyzing the first step of the pentose phosphate pathway in the cytosol [33] and endoplasmic reticulum [34], respectively, thereby linking metabolic status with antioxidant capacity [35].

In addition to the glutathione system, other antioxidant enzymes such as catalase and superoxide dismutase (SOD) are integral to the cellular antioxidant defense network [36]. Catalase rapidly decomposes hydrogen peroxide into water and oxygen, preventing its accumulation and the generation of more reactive species [27]. SOD catalyzes the dismutation of superoxide radicals into hydrogen peroxide, which is then detoxified by catalase and GPx [27].

The regulation of these antioxidant defenses is controlled by the nuclear factor erythroid 2–related factor 2 (Nrf2) pathway, a key transcription factor that modulates the expression of a wide range of antioxidant and cytoprotective genes [37,38]. Under basal conditions, Nrf2 is sequestered in the cytoplasm by Kelch-like ECH-associated protein 1 (KEAP1), which facilitates its ubiquitination and subsequent proteasomal degradation [37,38]. In response to oxidative stress, Nrf2 dissociates from KEAP1, translocates to the nucleus, and activates the transcription of genes involved in antioxidant defenses, including those encoding for GSH synthesis, GPx, GR, catalase, and SOD [37,38].

Given the central role of the Nrf2-KEAP1 axis in regulating antioxidant defenses, dysregulation of this pathway has been implicated in various pathological conditions, including cancer, neurodegenerative diseases, and cardiovascular disorders, where oxidative stress is a common underlying factor [39,40].

Considering the close relationship between inflammaging, oxidative damage accumulation, and aging [41], this study analyzed antioxidant defenses and oxidative damage in CCS compared to healthy age-matched and elderly subjects. All experiments were performed using MNCs isolated from peripheral blood (PB) because they are considered an excellent model for evaluating the health status of an entire organism [21,42]. Results show that CCS-MNCs are characterized by a reduction in antioxidant enzyme expression and activity due to lower Nrf2 expression compared to the healthy control samples, leading to oxidative damage to lipids, DNA, and proteins compared to controls. Notably, these effects were reversed by N-acetylcysteine (NAC) treatment, suggesting a possible development of targeted interventions that could mitigate the long-term impact of cancer treatment and improve the overall CCS QoL.

## 2. Materials and Methods

### 2.1. Reagents

All chemicals were sourced from Sigma Aldrich (St. Louis, MO, USA) unless specified otherwise. Ultrapure water (Milli-Q; Millipore, Billerica, MA, USA) was used consistently. All other reagents were of analytical grade.

### 2.2. Samples

All experiments were conducted using MNCs isolated from PB through a standard gradient-separation technique within 24 h of collection.

The study involved 149 childhood cancer survivors who had completed treatment, with ages ranging from 1 to 40 years. Among these, 74 had solid tumors (neuroblastoma 65%, Wilms’ tumor 8.7%, rhabdomyosarcoma 7.3%, Ewing sarcoma 2.6%, and other types 16.4%), while 75 had hematological malignancies (acute lymphoblastic leukemia 72%, Hodgkin’s lymphoma 10%, and acute myeloid leukemia 11.8%). All participants had undergone extended chemotherapy and/or radiotherapy as part of pediatric cancer clinical trials and were disease-free for at least one year at the time of the experimental analyses. The median time between the end of cancer treatment and blood sampling was 7.2 years, ranging from 1 to 25 years. Control groups included 70 age-matched healthy donors (ages 1 to 40 years) and 66 older subjects (ages 41 to 96 years) with no history of cancer (see Table 1). This study adhered to the principles outlined in the Helsinki Declaration and received approval from the University Research Ethics Committee (CERA) of the University of Genoa (approval number 2022/54). Written informed consent was obtained from all participants. For those under 18 years of age, consent was provided by a parent or legal guardian. The analyses were conducted without blinding.

### 2.3. N-Acetyl Cysteine (NAC) CCS-MNCs Treatment

As an attempt to improve the antioxidant defenses of CCS-MNCs, MNCs isolated from PB were cultured in RPMI medium supplemented with 10% FCS and 5% penicillin-streptomycin solution for 48 h in the presence or absence of 500 µM NAC [43]. Subsequently, the cells were collected, washed in PBS, and used for biochemical analyses.

### 2.4. Oxidative Phosphorylation (OxPhos) Efficiency Evaluation

To evaluate OxPhos efficiency, the ratio between the amount of ATP produced by FoF1 ATP synthase and the oxygen consumed by the electron transport chain (ETC) in the presence of pyruvate and malate as respiratory substrates was calculated, resulting in the P/O ratio. Under coupled conditions, where oxygen consumption is properly linked to ATP synthesis, this value typically reaches approximately 2.5. In contrast, under uncoupled conditions, the P/O ratio decreases proportionally to the extent of OxPhos inefficiency [44].

For the evaluation of ATP production and oxygen consumption rate (OCR), MNCs were permeabilized by incubation with 0.03 mg/mL digitonin for 10 min.

ATP synthesis was assessed using the luciferin/luciferase ATP bioluminescence assay kit CLSII (Roche, Basel, Switzerland) on a GloMax^®^ 20/20 Luminometer (Promega, Madison, WI, USA). The assay was conducted at 37 °C for 2 min, measuring ATP generated from added ADP. A total of 200,000 cells were added to the incubation medium (final volume 0.1 mL), which contained 10 mM Tris-HCl (pH 7.4), 50 mM KCl, 1 mM EGTA, 2 mM EDTA, 5 mM KH_2_PO_4_, 2 mM MgCl2, 0.6 mM ouabain, 0.040 mg/mL ampicillin, 0.2 mM p1p5-di(adenosine-5′) pentahosphate, 5 mM pyruvate, and 2.5 mM malate. After equilibrating the cells for 10 min at 37 °C, ATP synthesis was induced by the addition of 0.2 mM ADP and monitored for two minutes, with measurements taken every 30 s [45].

The OCR was determined using an amperometric oxygen electrode in a magnetically stirred, sealed chamber at 37 °C (Unisense, DK). Each assay used 200,000 cells suspended in a solution containing 137 mM NaCl, 5 mM KH_2_PO_4_, 5 mM KCl, 0.5 mM EDTA, 3 mM MgCl_2_, and 25 mM Tris–HCl (pH 7.4). The measurements were initiated with the addition of 5 mM pyruvate and 2.5 mM malate [45].

### 2.5. ROS Production Evaluation

To assess ROS levels, the ROS Detection assay kit (OZ Bioscience, Marseille, France, cod: OZBIROS0300) was used, following the manufacturer’s instructions, on freshly isolated MNCs resuspended in PBS. The fluorescence derived from the interaction between ROS and 2′,7′-dichlorodihydrofluorescein diacetate (H_2_DCFDA) was monitored with a PerkinElmer LS 50B fluorimeter (485 nm excit., 535 nm emiss.).

### 2.6. Oxidative Damage Accumulation Assay

To assess the accumulation of oxidative damage in MNCs, levels of malondialdehyde (MDA) and 4-hydroxynonenal (4-HNE) were measured as markers of lipid peroxidation, 8-hydroxy guanosine (8-OHdG) as a marker of oxidative DNA damage, and nitrotyrosine as a marker of protein oxidation. MDA levels were determined using the thiobarbituric acid reactive substances (TBARS) assay. The TBARS solution consisted of 15% trichloroacetic acid in 0.25 N HCl and 26 mM thiobarbituric acid. For basal MDA assessment, 600 μL of the TBARS solution was mixed with 50 μg of total protein in 300 μL Milli-Q water. The mixture was incubated at 95 °C for 40 min. After incubation, the sample was centrifuged at 14,000 rpm for 2 min, and the absorbance of the supernatant was measured spectrophotometrically at 532 nm [46].

Lipid Peroxidation (4-HNE) Assay Kit (cod: ab287803), 8-hydroxy-2′-deoxyguanosine (8-OHdG) ELISA Kit (cod: ab201734), and Nitrotyrosine ELISA Kit (cod: ab 116691) (all from AbCam) were used to evaluate the intracellular concentrations of 4-HNE, 8-OHdG, and nitrotyrosine, respectively, following the manufacturer’s instructions. For each assay, 50 μg of total protein was used.

### 2.7. Evaluation of NADPH, NADP, GSH, and GSSG Intracellular Concentration

NADP/NADPH Quantitation Kit (cod: MAK038, Merck, Darmstadt, Germany) and Glutathione GSH/GSSG Assay Kit (cod: MAK440, Merck, Darmstadt, Germany) were used to evaluate the intracellular concentration of NADPH, NADP, GSH, and GSSG, following the manufacturer’s instructions. For each assay, 50 μg of total protein was used.

### 2.8. Antioxidant Enzymes Activity Assay

For each assay, 50 μg of total protein was utilized.

G6PD activity was assayed spectrophotometrically at 340 nm, following the NADP reduction. The assay mix contained 100 mM Tris HCl, 5 mM MgCl_2_, 80 mM glucose-6-phosphate, and 1 mM NADP [47]. For the H6PD assay, the reaction mixture was the same used for G6PD, in which G6P has been replaced with the same amount of 2-deoxyglucose-6-phosphate [47].

GR activity was assessed at 340 nm, following the NADPH oxidation. The assay was conducted in a medium composed of 100 mM Tris-HCl (pH 7.4), 1 mM EDTA, 5 mM GSSG, and 0.2 mM NADPH [48].

GPx activity was assayed following the decomposition of hydrogen peroxide (H_2_O_2_) at 240 nm, using an assay solution containing 100 mM Tris-HCl (pH 7.4), 5 mM H_2_O_2_, and 5 mM GSH. Since H_2_O_2_ is also a substrate of catalases, GPx activity is obtained by subtracting the result of this assay from the catalase activity values (for catalase assay, see below) [48].

SOD Assay Kit was used to evaluate the superoxide dismutase activity, following the manufacturer’s instructions (cod: ab65354, AbCam, Cambridge, UK).

CAT activity was determined spectrophotometrically by monitoring the H_2_O_2_ decomposition at 240 nm. The reaction mixture included 50 mM phosphate buffer (pH 7.0) and 5 mM H_2_O_2_ [48].

### 2.9. Western Blot Analysis

MNCs were lysed in phosphate buffer saline (PBS) supplemented with a protease inhibitor cocktail for mammalian cells (Sigma). Total protein concentration was quantified using the Bradford assay [49]. Proteins were separated on 4–20% polyacrylamide gels via SDS-PAGE and transferred to a nitrocellulose membrane (BioRad Laboratories, Hercules, CA, USA). The membrane was blocked for 1 h in PBS-0.15% Tween 20 (PBSt) containing 5% non-fat dry milk, followed by overnight incubation at 4 °C with primary antibodies targeting G6PD (1:1000, cod: ab124738, AbCam), H6PD (1:1000, cod: ab170895, AbCam), GR (1:2000, cod:62448, Cell Signaling Technology, Danvers, MA, USA), GPx (isoform 1, 1:2000, cod: 3286S, Cell Signaling Technology), SOD1 (1:2000, cod: 65778SF, Cell Signaling Technology), SOD2 (1:2000, cod: 13141S, Cell Signaling Technology), CAT (1:2000, cod: BK12980S, Cell Signaling Technology), Nrf2 (1:2000, cod: ab137550, AbCam), KEAP1 (1:2000, cod: 8047S, Cell Signaling Technology), and beta-actin (1:1000, cod: MA1-140, ThermoFisher, Waltham, MA, USA). All antibodies were diluted in PBSt.

After washing with PBSt, the membrane was treated with horseradish peroxidase-conjugated anti-rabbit or anti-mouse IgG secondary antibodies (1:10,000, BioRad Laboratories) and visualized using Clarity Western ECL Substrate (BioRad Laboratories). The bands were detected, and their density was quantified using an enhanced chemiluminescence system (Alliance 6.7 WL 20 M, UVITEC, Cambridge, UK) and UV1D software (UVITEC). The intensity of each band was normalized to actin levels on the same membrane. All WB replicates are reported in Supplementary Figure S1.

### 2.10. Statistical Analyses

Non-parametric methods were used to compare CCS samples with both age-matched healthy controls and elderly healthy subjects, specifically employing the Mann–Whitney U test. Statistical analyses were conducted using GraphPad Prism software, version 10.00 (GraphPad Software Inc., La Jolla, CA, USA). A *p*-value of less than 0.05 was considered statistically significant.

## 3. Results

### 3.1. The CCS-MNCs Exhibit Impaired Energy Metabolism Efficiency and Increased ROS Production, Leading to the Accumulation of Oxidative Damage

**Figure 1:**
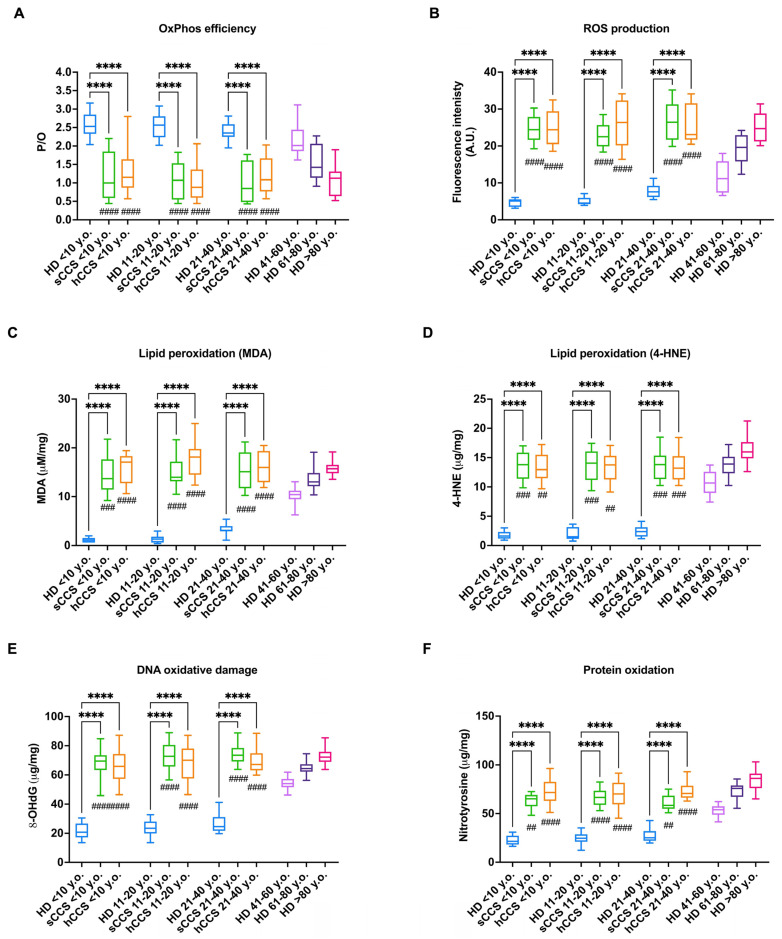
OxPhos efficiency, ROS production, and oxidative damage accumulation in MNCs isolated from CCS, age-matched healthy subjects, and elderly healthy controls. All data were obtained on MNCs isolated from age-matched healthy controls (<10 y.o., *n* = 18; 11–20 y.o., *n* = 32; 21–40 y.o, *n* = 20), CCS of solid tumor (sCCS; <10 y.o., *n* = 27; 11–20 y.o., *n* = 29; 21–40 y.o., *n* = 18), CCS of hematological tumors (hCCS; <10 y.o., *n* = 25; 11–20 y.o., *n* = 28; 21–40 y.o., *n* = 22), healthy donors aged between 41–60 y.o., *n* = 19; healthy donors aged between 61–80 y.o., *n* = 25; healthy donors aged over 80 y.o., *n* = 22. (**A**) P/O value, an OxPhos efficiency marker, evaluated in the presence of pyruvate plus malate as respiring substrates; (**B**) Reactive oxygen species (ROS) production; (**C**) Malondialdehyde (MDA) intracellular concentration, as a lipid peroxidation marker; (**D**) 4-hydroxynonenal (4-HNE) intracellular concentration, as a lipid peroxidation marker; (**E**) 8-Hydroxy-2′-deoxyguanosine (8-OHdG) intracellular concentration, as a DNA oxidative damage marker; (**F**) Nitrotyrosine intracellular level, as a protein oxidative damage marker. **** indicates a *p* < 0.0001. ##, ###, and #### indicate a *p* < 0.01, 0.001, or 0.0001, respectively, between sCCS or hCCS and healthy donors aged 41–60 y.o. No significant differences were observed between CCS samples and healthy subjects aged between 61–80 y.o. and over 80 y.o.

A previous study investigating metabolic changes in CCS samples found that MNCs isolated from these subjects exhibited mitochondrial metabolic defects, resulting in the accumulation of MDA [21]. This finding is corroborated in this new cohort of patients, where the P/O ratio, a well-established index of OxPhos efficiency, is significantly lower than 2.5 (the reference value observed when aerobic metabolism is stimulated with pyruvate plus malate in vitro), indicating that MNCs isolated from CCS, aged 0–40 years and treated for solid or hematologic tumors, are characterized by an uncoupling between mitochondrial energy synthesis and respiration. In contrast, this uncoupling is not observed in age-matched healthy subjects, where OxPhos remains properly coupled under the same conditions (Figure 1, panel A).

Additionally, the P/O values observed in MNCs isolated from CCS are similar to those of healthy subjects over 60 years old (Figure 1, panel A). This aerobic metabolism dysfunction is associated with increased ROS production, which is significantly higher in CCS compared not only to age-matched healthy subjects but also to individuals aged 40 to 60 years, while it is comparable to levels observed in healthy subjects over 60 (Figure 1, panel B). However, to assess whether the increased oxidative stress production caused cellular alterations that could justify the premature aging observed in CCS, several markers of oxidative damage were evaluated. As shown in Figure 1, panels C–F, CCS-MNCs are characterized by high levels of both MDA and 4-HNE, both markers of lipid peroxidation, as well as 8-OHdG and nitrotyrosine compared to the age-matched healthy control, reaching the levels observed in individuals over 60. This suggests that oxidative damage is also extended to DNA and proteins, in addition to lipids, as previously observed [21].

Interestingly, an examination of all parameters reported in Figure 1 reveals no significant differences between CCS-MNCs derived from patients with solid tumors compared to those derived from subjects with hematologic malignancy history. Moreover, the observed alterations are similar across the different decades of CCS analyzed. Finally, as reported in Supplementary Figure S1, the extent of damage does not change relative to the time elapsed between the end of therapy and sample analysis, allowing the assumption that the damage persists over time.

### 3.2. NADPH and GSH Intracellular Levels Are Reduced in CCS-MNCs

**Figure 2:**
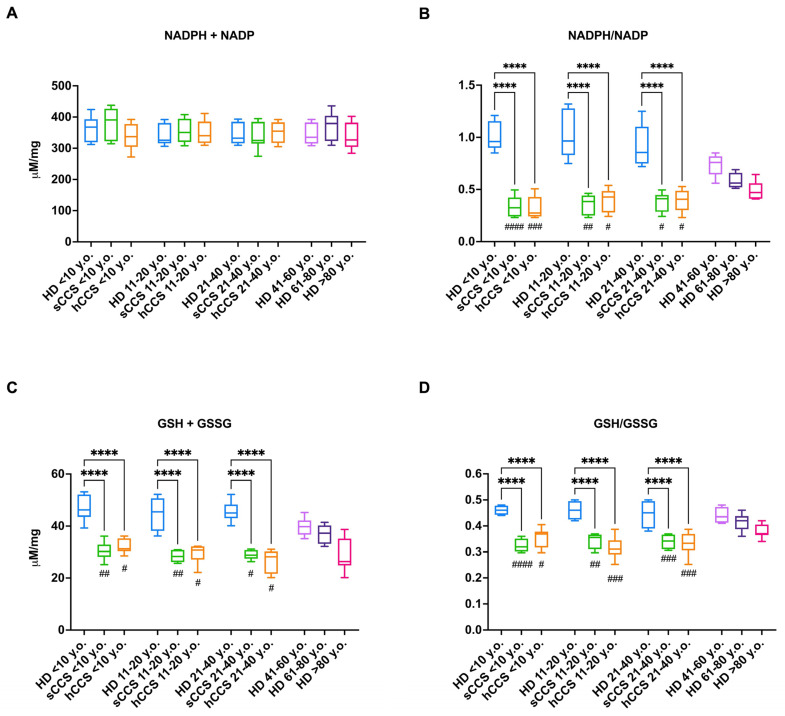
Intracellular level of oxidized and reduced forms of NADP and glutathione in MNCs isolated from CCS, age-matched healthy subjects, and elderly healthy controls. All data were obtained on MNCs isolated from age-matched healthy controls (<10 y.o., *n* = 18; 11–20 y.o., *n* = 32; 21–40 y.o., *n* = 20), CCS of solid tumor (sCCS; <10 y.o., *n* = 27; 11–20 y.o., *n* = 29; 21–40 y.o., *n* = 18), CCS of hematological tumors (hCCS; <10 y.o., *n* = 25; 11–20 y.o., *n* = 28; 21–40 y.o., *n* = 22), healthy donors aged between 41–60 y.o., *n* = 19; healthy donors aged between 61–80 y.o., *n* = 25; healthy donors aged over 80 y.o., *n* = 22. (**A**) Total intracellular concentration of reduced and oxidized forms of NADP; (**B**) Ratio between NADPH and NADP; (**C**) Total intracellular concentration of reduced and oxidized forms of glutathione (GSH + GSSG); (**D**) Ratio between GSH and GSSG. **** indicates a *p* < 0.0001. #, ##, ###, and #### indicate a *p* < 0.05, 0.01, 0.001, or 0.0001, respectively, between sCCS or hCCS and healthy donors aged 61–80 y.o. No significant differences were observed between CCS samples and healthy subjects over 80 y.o.

Given that the oxidative damage accumulation results from an imbalance between oxidative stress production and cellular antioxidant response, we evaluated the concentrations of NADP and GSSG, as well as their reduced forms, as they represent key molecules involved in cellular antioxidant defenses [29]. The results show that although the total amount of NADP (oxidized plus reduced forms) is similar across all analyzed samples (Figure 2, panel A), CCS-MNCs are characterized by significantly lower NADPH/NADP ratios compared to age-matched healthy subjects and healthy individuals over 60 years old, reaching a similar value observed in subjects aged over 80 (Figure 2, panel B). Regarding the oxidized and reduced forms of glutathione, it is evident that GSH/GSSG concentration follows a similar pattern to NADPH/NADP (Figure 2, panel D), being lower in CCS-MNCs compared to other samples; however, also the total glutathione amount appeared decreased (Figure 2, panel C), suggesting that CCS produce less glutathione than healthy age-matched subjects.

As with the parameters reported in Figure 1, no differences are observed between the different age groups of CCS analyzed, nor is there any improvement or deterioration relative to the time elapsed between the end of therapy and sample analysis (Supplementary Figure S2).

### 3.3. Oxidative Damage in CCS-MNCs Is Due to Reduced Expression and Activity of Enzymes Involved in Cellular Antioxidant Defenses

**Figure 3:**
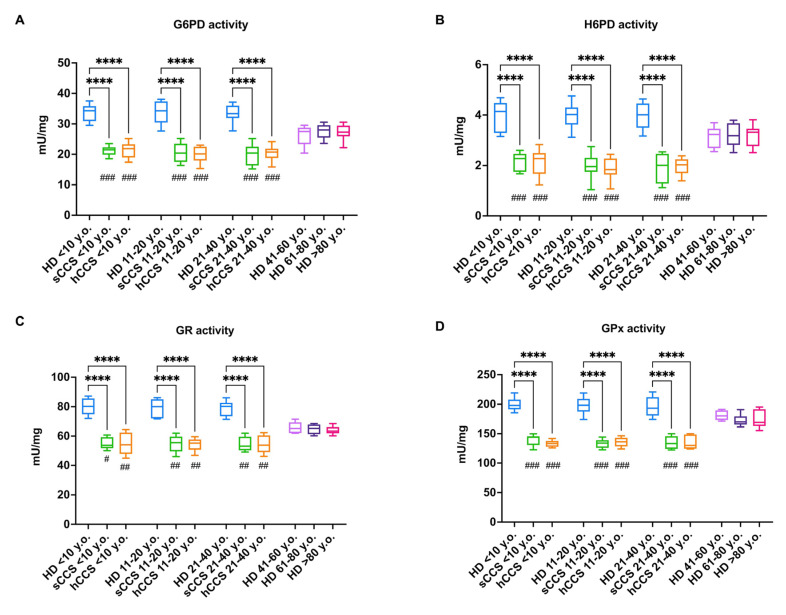

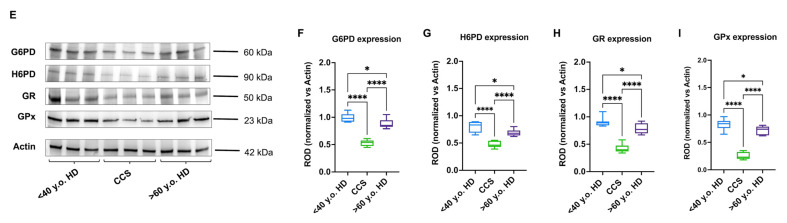
Activity and protein expression of G6PD, H6PD, GR, and GPX in MNCs isolated from CCS, age-matched healthy subjects, and elderly healthy controls. Data reported in Panels (**A**–**D**) were obtained on MNCs isolated from age-matched healthy controls (<10 y.o., *n* = 18; 11–20 y.o., *n* = 32; 21–40 y.o., *n* = 20), CCS of solid tumor (sCCS; <10 y.o., *n* = 27; 11–20 y.o., *n* = 29; 21–40 y.o., *n* = 18), CCS of hematological tumors (hCCS; <10 y.o., *n* = 25; 11–20 y.o., *n* = 28; 21–40 y.o., *n* = 22), healthy donors aged between 41–60 y.o., *n* = 19; healthy donors aged between 61–80 y.o., *n* = 25; healthy donors aged over 80 y.o., *n* = 22. Data reported in Panels (**E**–**I**) are representative of three independent experiments, each on *n* = 3 age-matched healthy donors (<40 y.o.); *n* = 3 (CCS < 40 y.o.), and *n* = 3 elderly healthy subjects (>60 y.o.) (**A**) G6PD activity; (**B**) H6PD activity; (**C**) GR activity; (**D**) GPx activity; (**E**) Representative Western blot signals of G6PD, H6PD, GR, and GPX. Actin was evaluated as a housekeeping signal. (**F**) Densitometric analysis of G6PD signal normalized against actin signal; (**G**) Densitometric analysis of H6PD signal normalized against actin signal; (**H**) Densitometric analysis of GR signal normalized against actin signal; (**I**) Densitometric analysis of GPx signal normalized against actin signal. ROD = Relative Optical Density. *, **** indicate a *p* < 0.05, 0.0001. In panels (**A**–**D**), #, ##, and ### indicate a *p* < 0.05, 0.01, or 0.001, respectively, between sCCS or hCCS and healthy donors over 80 y.o.

To determine whether the lower levels of NADPH and GSH depend on a defect in the activation of cellular antioxidant defenses, the activity and expression of several enzymes involved in the glutathione pathway and ROS detoxification were assessed. CCS-MNCs exhibit low activity levels of G6PD and H6PD (Figure 3, panels A and B, respectively), both involved in NADPH production [50], as well as of GR and GPx (Figure 3, panels C and D), enzymes responsible for glutathione reduction and employment as an antioxidant molecule, respectively [30]. Notably, the activities of these four enzymes in CCS-MNCs are lower compared to age-matched healthy samples and MNCs isolated from individuals over 40 years old across all decades evaluated. The defect in activity appears to be due to reduced enzyme expression, as indicated by the Western blot signals shown in Figure 3, panels E–I.

**Figure 4:**
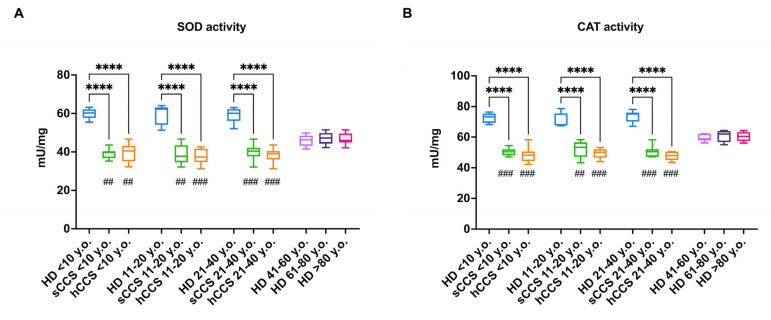

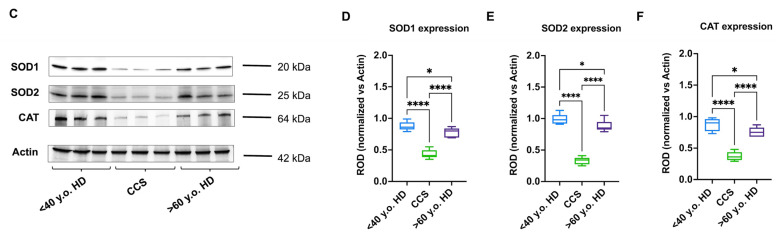
Activity and protein expression of SOD and CAT in MNCs isolated from CCS, age-matched healthy subjects, and elderly healthy controls. Data reported in Panels (**A**,**B**) were obtained on MNCs isolated from age-matched healthy controls (<10 y.o., *n* = 18; 11–20 y.o., *n* = 32; 21–40 y.o., *n* = 20), CCS of solid tumor (sCCS; <10 y.o., *n* = 27; 11–20 y.o., *n* = 29; 21–40 y.o., *n* = 18), CCS of hematological tumors (hCCS; <10 y.o., *n* = 25; 11–20 y.o., *n* = 28; 21–40 y.o., *n* = 22), healthy donors aged between 41–60 y.o., *n* = 19; healthy donors aged between 61–80 y.o., *n* = 25; healthy donors aged over 80 y.o., *n* = 22. Data reported in Panels (**C**–**F**) are representative of three independent experiments, each on *n* = 3 age-matched healthy donors (<40 y.o); *n* = 3 (CCS < 40 y.o.), and *n* = 3 elderly healthy subjects (>60 y.o.). (**A**) SOD activity; (**B**) CAT activity; (**C**) Representative Western blot signals of SOD1 (cytosolic form), SOD2 (mitochondrial form), and CAT. Actin was evaluated as a housekeeping signal. (**D**) Densitometric analysis of SOD1 signal normalized against actin signal; (**E**) Densitometric analysis of SOD2 signal normalized against actin signal; (**F**) Densitometric analysis of CAT signal normalized against actin signal. ROD = Relative Optical Density. *, **** indicate a *p* < 0.05, 0.0001. In panels (**A**,**B**), ##, and ### indicate a *p* < 0.01, or 0.001, respectively, between sCCS or hCCS and healthy donors over 80 y.o.

Since the glutathione pathway does not represent the only cellular system for oxidative stress detoxification, the activity and expression of SOD and CAT were also evaluated (Figure 4). Specifically, the data indicate that CCS-MNCs show a decrease in SOD and CAT activity compared to both age-matched and elderly individuals (Figure 4, panels A and B, respectively). Again, this defect appears to be due to reduced expression of both SOD1 (cytosolic form) and SOD2 (mitochondrial form) as well as CAT (Figure 4, panels C–F).

### 3.4. The Reduced Antioxidant Response in CCS-MNCs Is Due to Decreased Nrf2 Expression

**Figure 5:**
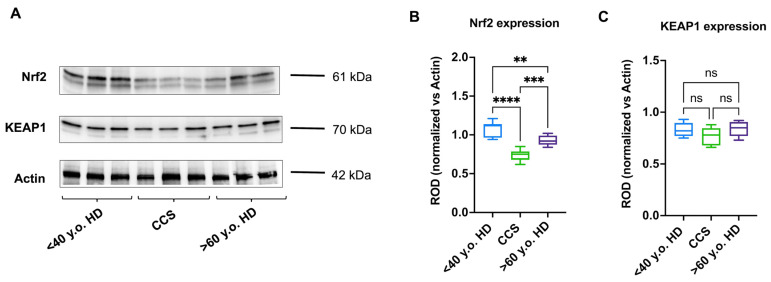
Nrf2 and KEAP1 protein expression in MNCs isolated from CCS, age-matched healthy subjects, and elderly healthy controls. Data reported in this figure are representative of three independent experiments, each on *n* = 3 age-matched healthy donors (<40 y.o); *n* = 3 (CCS < 40 y.o.), and *n* = 3 elderly healthy subjects (>60 y.o.). (**A**) Representative Western blot signals of Nrf2 and KEAP1. Actin was evaluated as a housekeeping signal. (**B**) Densitometric analysis of Nrf2 signal normalized against actin signal; (**C**) Densitometric analysis of KEAP1 signal normalized against actin signal. ROD = Relative Optical Density. **, ***, **** indicate a *p* < 0.01, 0.001, 0.0001. ns: not significant.

To understand the underlying mechanism responsible for the reduced expression and consequent activity of G6PD, H6PD, GR, GPx, SOD, and CAT, the expression levels of Nrf2 and its negative regulator, KEAP1, were evaluated, since the balance between these two proteins modulates the cellular capacity to enhance antioxidant defenses in response to increased oxidative stress [38]. The data show that CCS-MNCs exhibit lower levels of Nrf2 compared to age-matched and elderly samples (Figure 5, panels A and B), while KEAP1 levels appear similar across all analyzed samples (Figure 5, panels A and C). Interestingly, MNCs from healthy subjects over 60 show a higher expression of antioxidant enzymes (Figure 3 and Figure 4) and Nrf2 (Figure 5) compared to CCS-MNCs. However, the expression of these proteins is lower when compared to healthy controls under 40. These data confirm that antioxidant defenses and their regulatory mechanisms decrease during physiological aging [23].

### 3.5. NAC Treatment Enhances Nrf2 Levels and Consequently Activates Antioxidant Defenses in CCS-MNCs, Improving OxPhos Efficiency, Reducing ROS Production, and Mitigating Oxidative Damage

**Figure 6:**
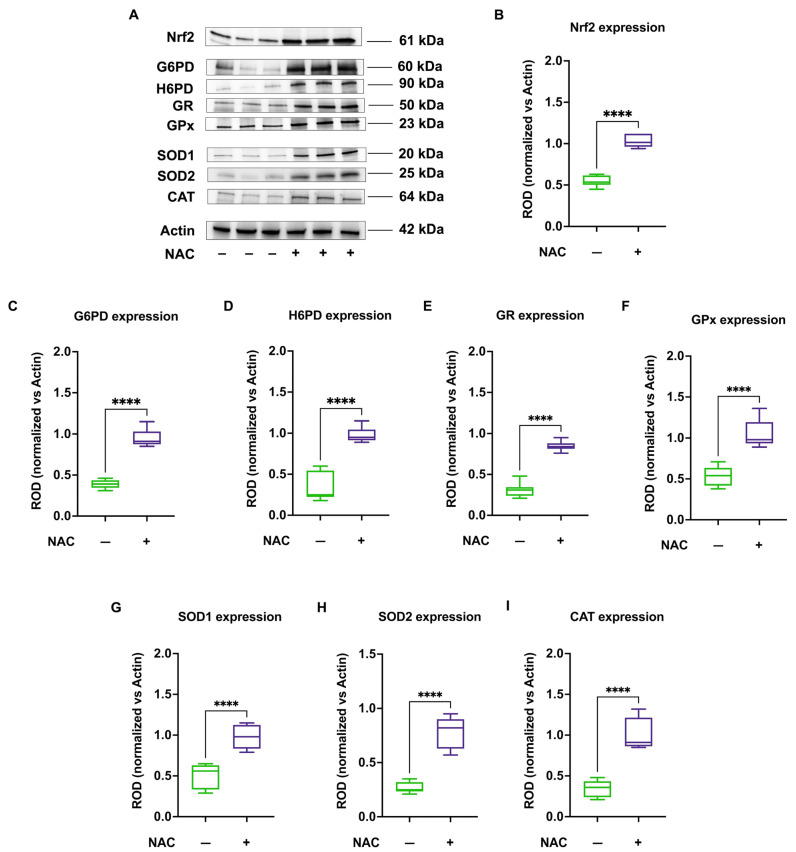
Nrf2 and antioxidant enzymes protein expression in MNCs isolated from CCS before and after NAC treatment. Data reported in this figure are representative of three independent experiments, each on *n* = 3 CCS and *n* = 3 same CCS treated with NAC. (**A**) Representative Western blot signals of Nrf2, G6PD, H6PD, GR, GPx, SOD1, SOD2, and CAT. Actin was evaluated as a housekeeping signal. (**B**) Densitometric analysis of Nrf2 signal normalized against actin signal; (**C**) Densitometric analysis of G6PD signal normalized against actin signal; (**D**) Densitometric analysis of H6PD signal normalized against actin signal; (**E**) Densitometric analysis of GR signal normalized against actin signal; (**F**) Densitometric analysis of GPx signal normalized against actin signal; (**G**) Densitometric analysis of SOD1 (cytosolic form) signal normalized against actin signal; (**H**) Densitometric analysis of SOD2 (mitochondrial form) signal normalized against actin signal; (**I**) Densitometric analysis of CAT signal normalized against actin signal. ROD = Relative Optical Density. **** indicates a *p* < 0.0001.

To induce antioxidant responses, CCS-MNCs were cultured for 48 h in the presence of 2 mM NAC, a molecule known to elevate Nrf2 levels. The results show that NAC treatment led to an increase in Nrf2 levels in CCS-MNCs (Figure 6, panels A and B), which, in turn, induced a significant increase in protein levels of the key enzymes involved in the antioxidant responses: G6PD, H6PD, GR, GPx, SOD1, SOD2, and CAT (Figure 6, panels A,C–I).

**Figure 7:**
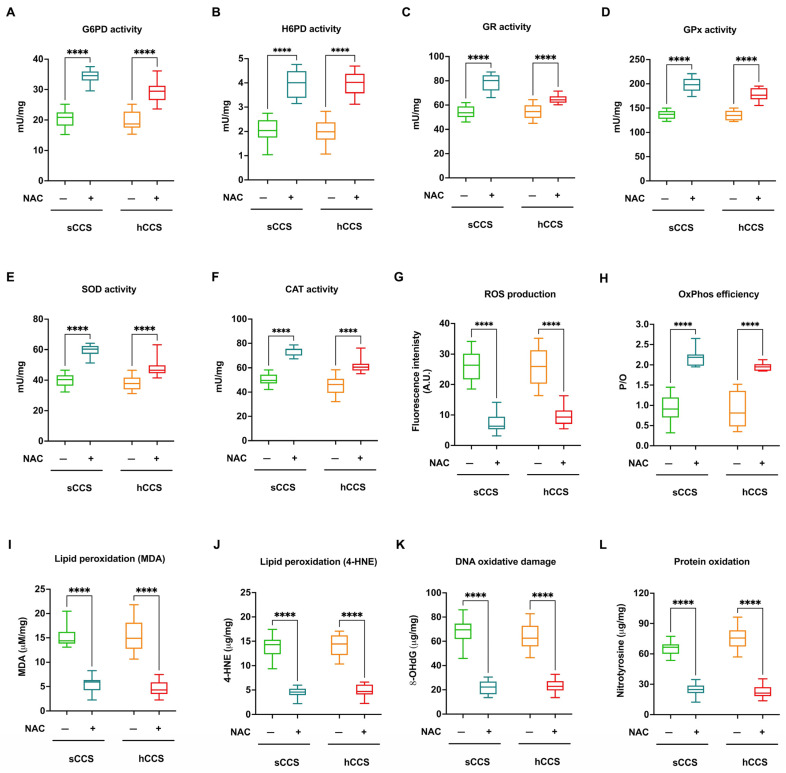
Antioxidant enzyme activities, OxPhos coupling, and oxidative damage accumulation in MNCs isolated from CCS before and after NAC treatment. Data reported in this figure were obtained on MNCs isolated from *n* = 20 CCS of solid tumor (sCCS; aged under 40 y.o.) and *n* = 22 CCS of hematological tumors (hCCS; aged under 40 y.o.) treated or not with NAC. (**A**) G6PD activity; (**B**) H6PD activity; (**C**) GR activity; (**D**) GPx activity; (**E**) SOD activity; (**F**) CAT activity; (**G**) ROS production; (**H**) P/O value as an OxPhos efficiency marker; (**I**) Malondialdehyde (MDA) intracellular concentration, as a lipid peroxidation marker; (**J**) 4-hydroxynonenal (4-HNE) intracellular concentration, as a lipid peroxidation marker; (**K**) 8-Hydroxy-2′-deoxyguanosine (8-OHdG) intracellular concentration, as a DNA oxidative damage marker; (**L**) Nitrotyrosine intracellular level, as a protein oxidative damage marker. **** indicates a *p* < 0.0001.

This increased expression translated into enhanced activity of these enzymes, contributing to an improved antioxidant response (Figure 7, panels A–F). Furthermore, NAC treatment reduced ROS production (Figure 7, panel G) and improved OxPhos coupling (Figure 7, panel H), resulting in decreased oxidative damage to lipids (Figure 7, panels I and J), DNA (Figure 7, panel K), and proteins (Figure 7, panel L).

## 4. Discussion

The data presented in this study demonstrate that MNCs isolated from CCS patients exhibit a defective antioxidant response, which, due to its inability to counterbalance the increased oxidative stress, leads to the accumulation of oxidative damage. This damage may contribute to the development of inflammaging and the premature aging associated with chemo/radiotherapy.

Based on the results reported in this work, the redox imbalance in CCS MNCs is due to the disequilibrium between two factors: (1) the increase in oxidative stress and (2) the inability to upregulate the expression of antioxidant defenses.

The observed increase in oxidative stress in CCS appears to be driven by the OxPhos uncoupling, which is sometimes associated with ROS production [51], confirming the findings of a previous study on metabolic dysfunctions in CCS [21]. It is well established that OxPhos is a major source of cellular ROS [52]. Mild uncoupling can reduce ROS production, impacting cellular aging [53]; by contrast, excessive uncoupling, as in the case of added uncoupling molecules, leads to increased superoxide production [54,55]. The P/O ratios evaluated in the presence of pyruvate and malate show that CCS MNCs exhibit a 60% decrease (median P/O around 1) compared to healthy, age-matched populations (median P/O around 2.5), indicating a pronounced uncoupling that may account for the ROS increase. However, the heightened oxidative stress in CCS may also be due to mitochondrial NADPH depletion, which could facilitate mitochondrial transition pore opening and the consequent cytochrome c release and oxidative stress production increment when membrane potential decreases in damaged mitochondria [56]. Additionally, since the degree of uncoupling observed in CCS-MNCs is comparable to that in healthy individuals over 60, it is possible to confirm that CCS are affected by early aging.

Typically, cells can increase their antioxidant defenses in response to heightened oxidative stress to prevent damage to cellular structures [26,27,36]. However, this capacity diminishes with age [23], as evidenced by the reduced expression and activity of G6PD, H6PD, GR, GPx, SOD, and CAT in over-60 healthy individuals compared to the younger. This age-related decline in antioxidant defenses agrees with the oxidative stress theory of aging [25]. Conversely, despite the increased ROS production in CCS-MNCs, there is no corresponding increase in the expression and activity of antioxidant enzymes, as already observed in the previous CCS cohort by proteomic analysis [21], suggesting that these cells cannot activate an adaptive response. Interestingly, the expression and activity levels of antioxidant enzymes in CCS-MNCs are lower compared to both age-matched healthy controls and elderly individuals, including those over 80 years old, suggesting that the early aging process in CCS patients may involve mechanisms distinct from physiological ones. One possible cause of the impaired antioxidant response in CCS may be the low levels of Nrf2, as its expression in CCS patients is lower than in age-matched and older control subjects. In fact, Nrf2 plays a pivotal role in the response to oxidative stress, as it is a transcription factor for antioxidant enzymes [39] that should increase in response to ROS production [38]. Additionally, the KEAP1 protein expression, which negatively regulates Nrf2, remains unchanged in CCS-MNCs compared to other samples. These findings suggest an additional negative effect on the Nrf2 function as, under basal conditions, Nrf2 is sequestered in the cytoplasm by KEAP1, which promotes its ubiquitination and subsequent proteasomal degradation. Therefore, it is plausible to speculate that the antioxidant pathways in CCS-MNCs are defective due to a reduced absolute expression of Nrf2 and an imbalanced Nrf2/KEAP1 ratio favoring the inhibitor. In addition, the literature reports that CCS-MNCs express significantly low levels of SIRT6 [21], a deacetylase involved in the regulation of the cellular redox state as a coactivator of Nrf2 [57]. It is also noteworthy that both the glutathione pathway and the pathways involving SOD and CAT are under the control of Nrf2, and, consequently, its impairment deprives the cell of these protective mechanisms. In light of these results, the reduction in NADPH and GSH levels observed in CCS-MNCs should be interpreted differently from what is seen in healthy individuals aged 60–80 years and those over 80. In CCS patients, the decreased levels of NADPH and GSH are primarily attributed to the low expression and activity of antioxidant enzymes and the inability to increase their levels due to diminished Nrf2 expression. Conversely, in elderly individuals, the lower levels of NADPH and GSH compared to younger healthy individuals are not due to reduced production, but rather to their increased use in counteracting the physiological rise in oxidative stress. Additionally, dysfunctional OxPhos forces cells to shift towards anaerobic metabolism to compensate for losses in aerobic ATP production. This shift results in an increased glycolytic flux, bypassing the pentose phosphate pathway and further decreasing the availability of NADPH.

Regarding oxidative damage accumulation, the data in this study demonstrate that the inability of CCS-MNCs to neutralize oxidative stress affects various cellular structures—lipids, proteins, and nucleic acids—compromising several functions. In particular, lipid peroxidation can cause alterations in the plasma membrane and the other organelles’ membranes, altering cell homeostasis and causing cell death [58]. Proteins, upon oxidation, can lose their three-dimensional conformation and, therefore, their function, accumulating within the cell and perpetuating the damage [59]. The oxidative damage to DNA could favor a higher predisposition to accumulating mutations, which, in turn, could increase the risk of developing a second tumor [60]. Furthermore, it is interesting to consider that the inner mitochondrial membrane that houses the respiratory complexes and the Fo-F1 ATP synthase could be one of the primary targets of excessive ROS production, triggering a vicious cycle, as damage to the membrane would further uncoupled respiration from energy synthesis, thereby increasing oxidative stress production.

From a biological perspective, the uncoupling of OxPhos, increased ROS production, and altered antioxidant defenses appear similar when comparing CCS with hematological malignancies to those with solid tumors, despite potential clinical heterogeneity within the CCS population. This data homogeneity suggests that the damage is independent of the cancer types and the therapeutic protocol employed. The alterations seem consistent across different decades of the analyzed CCS, indicating that the metabolic alterations are unrelated to the age at which patients underwent treatment. In addition, cellular damage remains constant over time, suggesting permanent damage to hematopoietic stem cells (HSCs). The probable causes of the continuing damage could depend on the simultaneous activity of HSCs during the chemo/radiotherapy treatments, although the literature still debates this point [61,62], or because HSCs may maintain a metabolic memory following epigenetic modifications induced by the damage [63]. The damage caused by chemotherapy/radiotherapy may lead to alterations in DNA methylation, histone post-translational modifications (PTMs), non-coding RNAs (ncRNAs), as well as changes in circular RNAs [64], and RNA methylation, specifically affecting N6-methyladenosine [65], thereby influencing HSC metabolism in the long term. Indeed, Chen and Natarajan have observed that epigenetic changes during diabetes persist despite glucose normalization, driving uncontrolled oxidative stress, inflammation, fibrosis, and other pathological phenotypes [63]. Furthermore, the same authors suggest that HSCs, like immune and peripheral cells, concur in triggering metabolic memory [63]. However, further investigations are needed to fully understand the upstream and downstream mechanisms driving these persistent epigenetic variations.

Finally, the results show that treatment with NAC, an antioxidant molecule and precursor of glutathione [66], improves the Nrf2 expression and, consequently, all downstream antioxidant defenses. On the other hand, the literature reports that NAC is a positive Nrf2 modulator [67,68], although the mechanism remains unclear. A potential explanation may involve the Keap1 sulfhydration that causes the Nrf2 activation due to the increased production of H_2_S associated with NAC treatment [69,70]. Indeed, it has been proposed that NAC exerts its antioxidant effects through various mechanisms, including cysteine desulfuration, which releases hydrogen sulfide and sulfane sulfur species, which display cytoprotective effects on cells [71]. The increased expression of antioxidant enzymes leads to a reduction in oxidative damage which, in turn, decreases ROS production and improves OxPhos coupling, confirming the association between metabolic alterations and oxidative damage accumulation in CCS-MNCs.

### Limitations of the Study

The study described in this manuscript represents preclinical research aimed at identifying one of the potential mechanisms underlying premature aging in CCS individuals. Accordingly, the use of NAC in this study does not imply an immediate clinical application, as it has been employed only to confirm that the oxidative damage accumulation was due to low antioxidant defenses caused by reduced Nrf2 expression, which NAC can enhance. Thus, although the data suggest that the antioxidant molecule administration could represent a possible treatment to reduce or slow the premature aging process in CCS individuals, we are currently unable to identify the optimal molecules, dosages, or methods and timing of administration. Further studies will be necessary before antioxidant agents can be applied in CCS individuals to prevent early aging.

Considering that aging depends on multiple factors, including lifestyle, comorbidities, and genetic predispositions, we acknowledge that the biochemical outcomes analyzed could have been influenced by factors unrelated to chemo/radiotherapy exposure. However, we can confirm that no healthy subjects, whether age-matched to the CCS group or over 40, presented any comorbidities at the time of sampling. Regarding lifestyle, approximately 20% of healthy subjects over 20 years of age were smokers, and 10% were overweight, whereas all CCS individuals were of normal weight and adhered to a healthy lifestyle due to their previous clinical condition. Unfortunately, we do not have information regarding the genetic predispositions of the analyzed subjects. On the other hand, we would point out that, despite these potential confounding factors, the results show a significant difference in mitochondrial function and antioxidant defenses of CCS MNC compared to age-matched and elderly control subjects.

## 5. Conclusions

Although the early fragility of CCS may depend on several molecular alterations, our data indicate that the inability to manage oxidative stress associated with energy metabolism alterations following chemo/radio treatments represents a key point of the early aging pathogenesis of CCS. Additionally, utilizing molecules capable of reversing oxidative damage may offer a promising strategy to target the molecular mechanisms that lead to the accumulation of cellular damage and, thus, potentially slow down the onset of chemo/radio therapy-related frailty in CCS.

## Figures and Tables

**Table 1:** Characteristics of CCS and control subjects.

**Childhood Cancer Survivors**					
	**Solid Tumors**			**Hematological Tumors**	
**Age (Years)**	**Subjects n°**	**Female/Male**		**Subjects n°**	**Female/Male**
<10	27	16/11		25	9/16
11–20	29	13/16		28	15/13
21–40	18	8/10		22	12/10
**Controls**					
	**Age-Matched**			**Adult**	
**Age (Years)**	**Subjects n°**	**Female/Male**	**Age (Years)**	**Subjects n°**	**Female/Male**
<10	18	7/11	41–60	19	8/11
11–20	32	18/14	61–80	25	15/10
21–40	20	11/9	>80	22	15/7

## Data Availability

Data are available on request from the authors.

## Supplementary Materials

The following supporting information can be downloaded at: https://www.mdpi.com/article/10.3390/antiox13111397/s1, Figure S1: Comparison of OxPhos efficiency, ROS production, and oxidative damage accumulation in MNCs isolated from CCS with respect to the time elapsed since therapy; Figure S2: Comparison of OxPhos efficiency, ROS production, and oxidative damage accumulation in MNCs isolated from CCS with respect to the time elapsed since therapy.
