# Supplementary material for: Redox Imbalance and Antioxidant Defenses Dysfunction: Key Contributors to Early Aging in Childhood Cancer Survivors
Source: Antioxidants (Basel). 2024 Nov 15;13(11):1397. doi: 10.3390/antiox13111397 (PMC11590913; doi:10.3390/antiox13111397)
Supplement: Supplementary file 1 [file antioxidants-13-01397-s001.zip › antioxidants-3302702-supplementary.pdf]

Supplementary Figure S1.

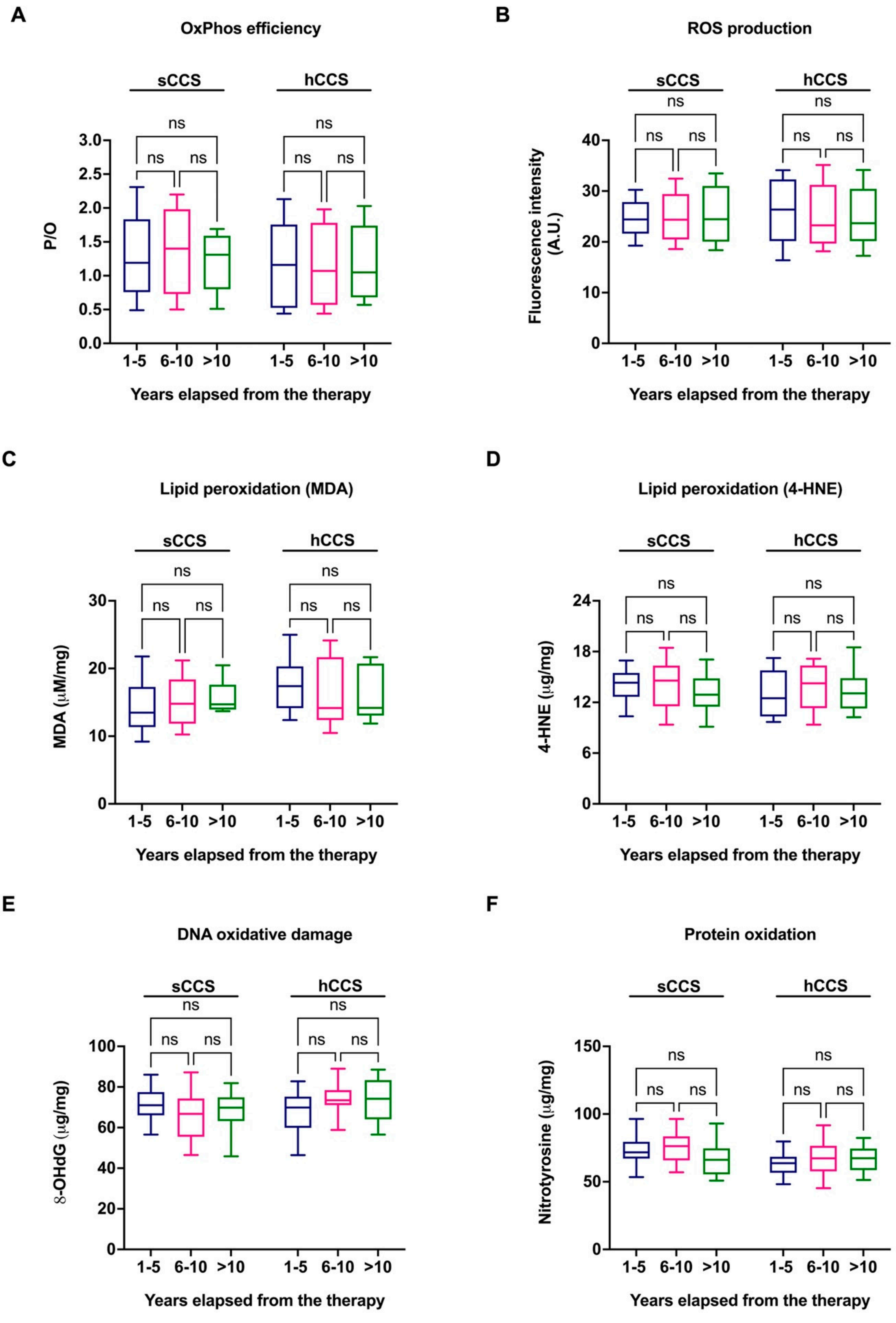

Supplementary Figure S1. Comparison of OxPhos efficiency, ROS production, and oxidative damage accumulation in MNCs isolated from CCS with respect to the time elapsed since therapy.

All data have been obtained on MNCs isolated from CCS of solid tumor (sCCS) who had been out of therapy for 1-5 years (n = 20), for 6-10 years (n = 38), for more than 10 years (n = 16) and from CCS of hematological tumor (hCCS) who had been out of therapy for 1-5 years (n = 27), for 6-10 years (n = 30), for more than 10 years (n = 18).

(A) P/O value in the presence of pyruvate plus malate as respiring substrates, as an OxPhos efficiency marker; (B) Reactive oxygen species (ROS) production; (C) Malondialdehyde (MDA) intracellular concentration, as a lipid peroxidation marker; (D) 4-hydroxynonenal (4-HNE) intracellular concentration, as a lipid peroxidation marker; (E) 8-Hydroxy-2'-deoxyguanosine (8-OHdG) intracellular concentration, as a DNA oxidative damage marker; (F) Nitrotyrosine intracellular level, as a protein oxidative damage marker. No significant differences were observed between the different groups based on the time elapsed since the last therapy and the metabolic analyses.

Supplementary Figure S2.

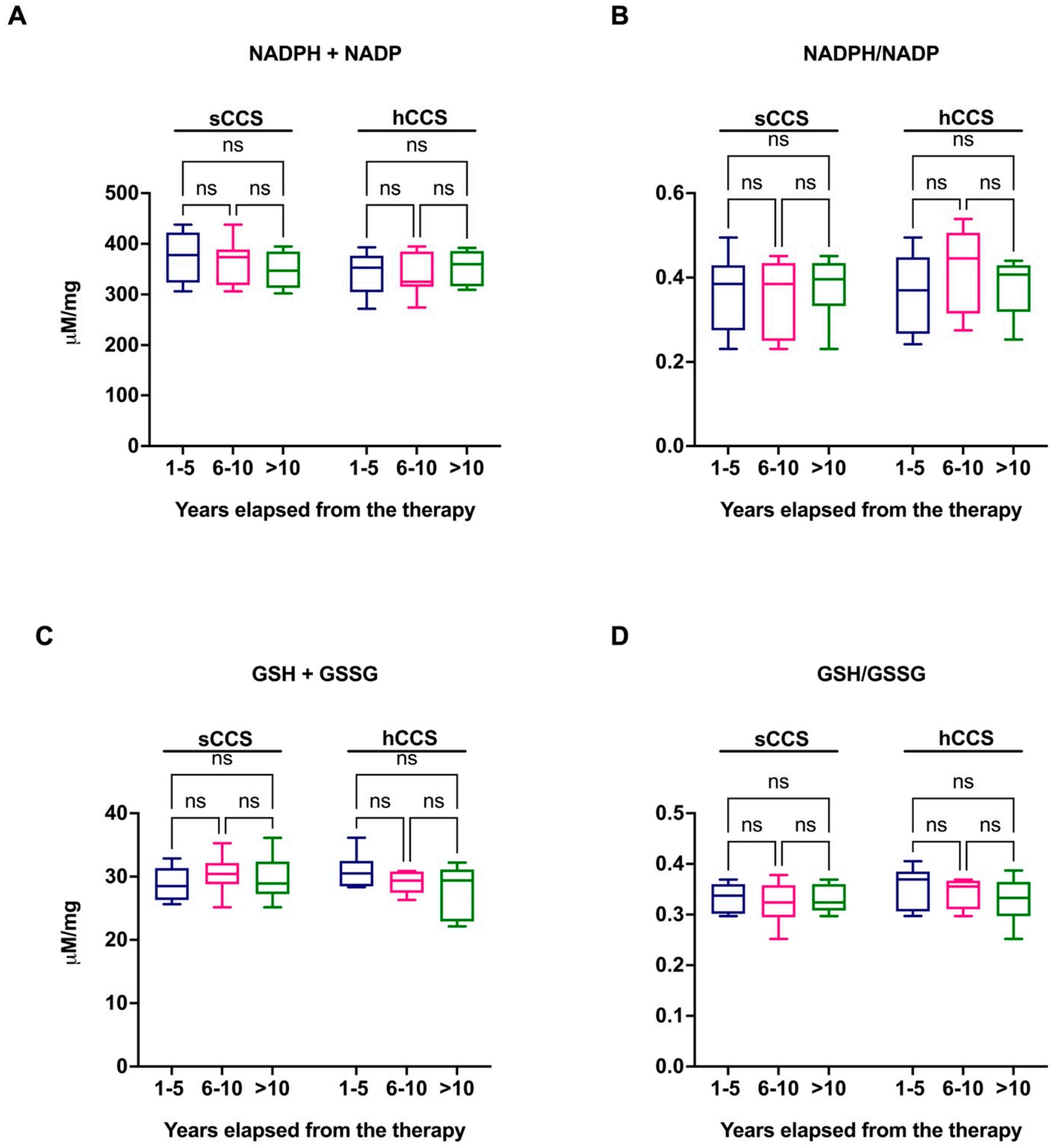

Supplementary Figure S2. Comparison of OxPhos efficiency, ROS production, and oxidative damage accumulation in MNCs isolated from CCS with respect to the time elapsed since therapy.

All data have been obtained on MNCs isolated from CCS of solid tumor (sCCS) who had been out of therapy for 1-5 years (n = 20), for 6-10 years (n = 38), for more than 10 years (n = 16) and from CCS of hematological tumor (hCCS) who had been out of therapy for 1-5 years (n = 27), for 6-10 years (n = 30), for more than 10 years (n = 18).

(A) Total intracellular concentration of reduced and oxidized forms of NADP; (B) Ratio between NADPH and NADP; (C) Total intracellular concentration of reduced and oxidized forms of glutathione (GSH + GSSG); (D) Ratio between GSH and GSSG. No significant differences were observed between the different groups based on the time elapsed since the last therapy and the metabolic analyses.
